# Experimental Design Based Optimization and Ex Vivo Permeation of Desmopressin Acetate Loaded Elastic Liposomes Using Rat Skin

**DOI:** 10.3390/pharmaceutics13071047

**Published:** 2021-07-09

**Authors:** Mohammad A. Altamimi, Afzal Hussain, Sultan Alshehri, Syed Sarim Imam

**Affiliations:** Department of Pharmaceutics, College of Pharmacy, King Saud University, P.O. Box 2457, Riyadh 11451, Saudi Arabia; maltamimi@ksu.edu.sa (M.A.A.); salshehri1@ksu.edu.sa (S.A.); simam@ksu.edu.sa (S.S.I.)

**Keywords:** elastic liposomes, desmopressin acetate, design expert based experimental design, ex vivo permeation profiles, hemocompatibility, vesicle–skin interaction

## Abstract

The study aimed to develop elastic-liposome-based transdermal delivery of desmopressin acetate for enhanced permeation to control enuresis, central diabetes insipidus, and traumatic injury. Elastic liposomes (ELs)-loaded desmopressin acetate was prepared, optimized, and evaluated for improved transdermal permeation profiles using rat skin. Full factorial design with independent factors (X_1_ for lipid and X_2_ for surfactant) at three levels was used against four responses (Y_1_, Y_2_, Y_3_, and Y_4_) (dependent variables). Formulations were characterized for vesicle size, polydispersity index (PDI), zeta potential, % entrapment efficiency (% EE), in vitro drug release, in vitro hemolysis potential, ex vivo drug permeation and drug deposition (DD), and ex vivo vesicle–skin interaction using scanning electron microscopy studies. The optimized formulation ODEL1 based on desirability function was found to have vesicle size, % EE, % DR, and permeation flux values of 118.7 nm, 78.9%, 75.1%, and 5.3 µg/h·cm^2^, respectively, which were close to predicted values. In vitro release profiles indicated slow and sustained delivery. Permeation flux values of ODEL1 and ODEL2 were 5.3 and 3.1 µg/h·cm^2^, respectively, which are 7.5- and 4.4-fold higher as compared to DS (0.71 µg/h·cm^2^). The obtained flux was relatively higher than the clinical target value of the drug for therapeutic efficacy. Moreover, the DD value of ODEL1 was significantly higher than ODEL2 and DS. Hemocompatibility study confirmed safety concerns. Finally, vesicle–skin interaction corroborated mechanistic views of permeation through rat skin. Conclusively, the transdermal delivery may be a suitable alternative to oral and nasal delivery to treat nocturnal enuresis, central diabetes insipidus, hemophilia A and von Willebrand’s disease, and any traumatic injuries.

## 1. Introduction

Desmopressin acetate is a synthetic potent analogue of vasopressin to treat nocturnal enuresis (young), central diabetes insipidus (DI), hemophilia A, von Willebrand’s disease before surgery (type I), and traumatic injuries. The drug is administered through various routes and doses, such as 20–40 µg (parenteral), 100–200 µg (oral), and 20–40 µg (nasal), respectively, which shows limited oral (<1%) and nasal (<3.4%) bioavailability due to enzymatic degradation (enzymes of gut lumen and nose mucosal tissues) and low lipophilicity of this peptide molecule [1,2]. Moreover, intranasal administration is associated with uncertainty in overdose level, inconvenient and unpredictable bioavailability, especially to those patients suffering with rhinorrhea (blocked nose), nasal hyperemia, and pediatric patients [3]. Commercially available (Octim^®^, DesmoMelt^®^, Stimate^®^, Minirin^®^) oral disintegration tablets (ODT) are an alternative to nasal products, with several advantages. However, these oral dosage forms are associated with various issues, such as (a) low lipophilicity of desmopressin acetate, (b) high molecular weight (1129 Da), and (c) small peptide, rendering the drug poorly absorbed from the gastrointestinal tract (GIT). Chemically, the drug has a disulfide bond and a cleavage site for α-chymotrypsin, which results in the peptide inactivation and, subsequently, leads to an extensive pre-systemic drug degradation after oral administration [1,2,4,5]. Thus, many attempts have been made to develop alternative oral formulations for enhanced bioavailability, such as solid lipid nanoparticles, mucoadhesive microemulsion, oral SEDDS (self-emulsifying drug delivery system), and prodrug synthesis [6,7,8]. Despite the parenteral formulation demonstrated better bioavailability, it is inconvenient for routine administration to young children.

Taking into account these contexts, transdermal delivery of desmopressin acetate may be an acceptable route of administration. Various physical methods (iontophoresis) have also been investigated for transdermal delivery of arginine–vesopressin [9,10,11]. Similarly, desmopressin acetate was administered transdermally using an iontophoresis technique across rat skin [12,13]. Cormier et al. studied transdermal delivery of desmopressin using a microneedle array (Macroflux^®^) technique to overcome the skin barrier. Authors reported 85% bioavailability with 30% variability, wherein only 10% of the loaded desmopressin was found to be on the applied site of skin [14]. However, these methods are expensive and arduous, with poor patient compliance.

From literature, it is well known that the impact of the drug on elastic liposomes is rarely taken into account in terms of permeation parameters and in vivo performance. However, permeation into or through the skin of a drug depends upon several factors, such as (a) size of a drug and carrier, (b) shape of carrier, (c) charge on drug and carrier, (d) polarity of a drug and carrier, (e) concentration and types of surfactant/lipid, (f) solubility of drug and surfactant, (g) HLB (hydrophilic lipophilic balance) value of the surfactant, (h) temperature, and (i) chemical nature of drug and excipients [15]. It is hard to predict, based on chemical structure and physical properties of a drug, whether the drug will permeate to the dermal area or not, and the rate and extent of permeation. Elastic liposomes contain three different environments to dissolve drug, such as (a) the lipophilic core for a lipophilic drug, (b) hydrophilic interior chamber for a water soluble drug (such as desmopressin acetate), and (c) the water–lipid interface for an amphiphilic molecule (for example, amphotericin B) [15]. Incorporation of a drug into elastic liposomes may change the membrane fluidity and, subsequently, penetration into the skin depending on molecular weight, formal change, partition coefficient, and solubility [15]. In the present study, the drug is associated with instability under acidic medium, poor oral bioavailability, substrate for proteinases, and aqueous solubility. Desmopressin is a small peptide (molecular weight ~1129 g/mole) associated with aqueous solubility of 2 mg/mL, zero formal charge, and negative log P (−6.2). Peralta et al. investigated improved penetration of water-soluble indole across mice skin regardless of molecular weight, as compared to complex and lipophilic amphotericin B [15]. Considering these, the drug may be maximally encapsulated in an aqueous interior environment of elastic liposomes [5]. Transdermal delivery may offer several advantages over oral delivery, such as (a) avoiding degradation from gastric acidic medium, (b) protecting the drug from enzymatic degradation, (c) it is targeted to systemic circulation by avoiding hepatic first pass metabolism, and has a controlled release profile. Law et al. demonstrated that the drug was stable (half-life ~177 h) at pH 4, even at high temperature, when tailored into desmopressin-loaded liposomes [16].

In general, liposomes comprised of phospholipid, cholesterol, and edge activator are considered to be confined to the uppermost epidermal region after topical application due to cholesterol imparted rigidity in the bilayer [17]. Several drugs have been explored for transdermal delivery using liposomes. However, liposomes exhibited limited permeation flux, low enhancement ratio, and were inefficient for systemic delivery. Therefore, elastic liposomes comprised of phospholipid, edge activator, and ethanol (7–10% *v*/*v*) are nanosized vesicles capable of accommodating challenges faced in conventional rigid liposomes, which are widely investigated for increased drug delivery topically or transdermally [18]. Moreover, elastic liposomes are cost effective (lack of cholesterol), ultra-deformable, have access to the dermal region of the skin, and are scalable for large-scale production [17]. The proposed carrier is biocompatible, safe, biodegradable, and well-explored for delivery of both hydrophilic and lipophilic drugs. Notably, elastic liposomes (ultra-deformable) are capable of responding to the external physical stress by promptly changing their shape, and penetrating the skin to the inner depth (dermis) for maximum drug access (systemic circulation) [19]. The carrier is suitable to deliver high molecular weight drug, peptides (such as desmopressin), and protect from degradation. Moreover, there have been several reports published for targeted delivery to the site of action, such as infection lesion area and tumor tissue [20]. Recently, several peptides (cell-penetrating peptides, CPPs) based functionalized elastic liposomes have been reported that facilitated enhanced transdermal drug delivery, achieved through peptide-mediated reversible changes in skin lipid [21,22,23]. Moreover, there have been various advancements in the therapeutic approach of elastic liposomes for transdermal and topical delivery of proteins and peptides so far. Thus, the drug is suitable for transdermal delivery using an elastic liposome for stable and controlled drug delivery to control nocturnal enuresis, central DI, and traumatic conditions. The study highlighted the optimization using experimental design tools, in vitro characterizations, and improved skin permeation profiles of the desmopressin-ferrying elastic liposome using a rat skin.

## 2. Materials and Methods

### 2.1. Materials

Desmopressin acetate (DA) was procured from Jinlan Pharm Drugs Technology Co., Limited (Hangzhou, China). Phospholipon^®^ 90G (P-90G), which is pure phosphatidylcholine soothed with 0.1% ascorbyl palmitate, was procured from Phospholipid GmbH (Nattermannallee 1, Koeln, Germany). Polysorbate 80 (Tween 80), Span 80, and Carbopol 934 (Carbopol) was obtained from Thermo Fisher Scientific (Waltham, MA, USA). Sodium cholate was obtained from Sigma-Aldrich (St. Louis, MO, USA). All other chemicals used were of analytical grade and Millipore water was used as an aqueous medium.

### 2.2. Analysis Methodology

To determine desmopressin concentration permeated across rat skin, high-performance liquid chromatography (HPLC) was used, employing a reverse phase C_18_ column (250 × 4.5 mm, 5 µ as particle size). The drug analysis was carried out as per methods reported previously [16]. The mobile phase was comprised of methanol, acetonitrile, and phosphate buffer (pH 7.0) in the ratio of 18:5:5 % *v*/*v*. The mobile phase mixture was freshly prepared and filtered through a membrane filter, followed by bath sonication to remove entrapped air bubbles. The analysis was progressed over a run time of 5 min, with an isocratic flow rate of 1 mL/min at room temperature. The drug was quantitatively estimated using a UV detector at 220 nm. A standard calibration curve was regressed with a regression coefficient (r^2^) of >0.999 over the concentration range of 0.1–80 µg/mL. The experiment was carried out in triplicate to obtain mean and standard deviation.

### 2.3. Preparation of Formulations

ELs were prepared by rotary evaporation technique (RET) with slight modification [24]. Phosphatidylcholine (PC) and surfactant were dissolved in a methanol–chloroform mixture (1:2). A total of nine formulations were prepared using three different surfactants, such as Tween-80 (DELT90, DELT70, and DELT60), Span 80 (DELS90, DELS70, and DELS60), and sodium cholate (SC) (DSC90, DSC70, and DSC60), at varied ratios (PC: surfactant). Three suitable ratios were 90:10, 70:30, and 60:40 for each surfactant. One liposome formulation was prepared for comparison purposes with a constant amount of the drug. In brief, a weighed amount of PC and surfactant was dissolved in a round bottom flask (RBF) containing 3 mL of methanol–chloroform mixture. Desmopressin acetate was accurately weighed (20 mg) and dissolved separately in a PBS solution (10 mL) to get a final concentration of 2 mg/mL. The final pH of PBS solution was adjusted to 5.5. The organic phase of RBF was rotated over a rotary evaporator (IKA, Staufen, Germany) at 50.0 rpm to remove organic solvent. The evaporation was fastened under reduced temperature (40 ± 2 °C) and pressure to generate a thin organic lipidic film on the inner wall. The developed film was hydrated with 10 mL of PBS (pH 5.5) containing DA (20.0 mg) and ethanol (7% *v/v*) [25]. In the case of liposome, the same procedure was repeated with slight modification. The organic phase was composed of PC, cholesterol (only for liposomes), and Span 80 in a fixed ratio (75:10:15). The aqueous hydrating solution was free from alcohol in the liposomes. Final formulations were sonicated for 60 s to reduce vesicle size. The obtained colloidal milky ELs were stored in a freezer overnight for activation. Each mL of formulation contained 2 mg of DA (0.2% *w/w*).

### 2.4. In Vitro Characterizations

Developed formulations were characterized for vesicle size, polydispersity index (PDI), elasticity, zeta potential, and % entrapment efficiency (% EE). Based on these studies, a suitable formulation was selected for further optimization using a Design Expert^®^ software (an optimization tool).

#### 2.4.1. Particle Size and Size Distribution, and Zeta Potential

All of the formulations were evaluated for vesicle size, PDI, and zeta potential using a Zetasizer Nano ZS (Malvern Instruments, Worcestershire, MA, USA) equipped with 4.0 mW He Ne red laser (633 nm) as per the reported method [18]. The sample was diluted with Millipore water before size analysis, whereas the zeta potential was measured using undiluted sample. The study was carried out at room temperature (25 °C) and a scattering angle of 90°.

#### 2.4.2. Elasticity of the Formulations

ELs are well known for ultra-deformability and elastic nature compared to conventional liposomes (rigid lipid bilayer due to cholesterol). Therefore, this nature was evaluated by an extrusion method [26]. The test formulation (10 mL) was allowed to pass through a membrane filter (micropore size of 50 nm less than the size of vesicles) [26]. The size of the extruded sample and the unpassed content was determined to calculate the elasticity (E) using Equation (1):

Elasticity (*E*) = *J* (R_v_/R_p_)^2^ (1)

where “*E*” and “*J*” are the elasticity and the volume (mL) extruded in 10 min, respectively. Similarly, R_v_ and R_p_ are the vesicle size after complete extrusion/the membrane aperture, respectively.

#### 2.4.3. Percent Entrapment Efficiency (% EE)

The prepared sample (10 mL) was centrifuged to obtain a vesicle pellet at the bottom. The supernatant was used for analysis of free DA using distilled water as a blank. The drug concentration was estimated using a UV–Vis spectrophotometer (Perkin-Elmer, Marlborough, MA, USA) at 220 nm. Percent entrapped drug was calculated using Equation (2):

Percent EE (% EE) = (A_t_ − A_s_)/At × 100 (2)

where “A_t_” and “A_s_” are the initial drug content loaded in the formulation and free drug available in the supernatant, respectively [27].

### 2.5. Optimization: Experimental Design Tool (Design Expert^®^)

Based on the preliminary findings (Table 1), the best formulation was selected and a full factorial design (3^2^) was applied to explore an optimized concentration of excipients, which may give a robust formulation. The software (Design Expert 7.0.0, Minneapolis, MN, USA) predicts several combinations under set conditions of goals. The experimental tool uses a random order at nine expected combinations. In this study, we used full factorial design (FFD), selecting two factors (X_1_ and X_2_) at three levels, such as minimum (−1), intermediate (0), and maximum (+1), represented as 3^2^ (level^factors^). This tool helps to identify the factors affecting an independent variable (responses) and omit non-significant factors during the optimization process. Factors and their levels were selected based on the preliminary findings (vesicle size, % EE, PDI, and elasticity). Thus, PC (X_1_) and SC (X_2_) were dependent variables against four independent variables (responses), which are vesicle size (Y_1_), % EE (Y_2_), % DR (Y_3_), and permeation flux (Y_4_). These dependent variables are vesicle size (Y_1_), (% EE) (Y_2_), % DR (Y_3_), and permeation flux (Y_4_). Both factors were selected based on the vesicle size (minimum), % EE, elasticity, and PDI values as shown in Table 1. Formulations comprised of PC, and SC were found to be fit as required for transdermal delivery. Reduced size may be permeated across the microscopic tiny pores of skin due to high elasticity, ultra-deformability, and membrane fluidity (under mild mechanical stress). However, an optimum concentration of PC and surfactant (SC) is required for successful delivery of desmopressin acetate (0.2% *w/w*) on transdermal application to achieve therapeutic level. Therefore, two levels of PC were selected as 200 (low) and 300 mg (high), whereas SC was set at 20 (low) and 120 mg (high). However, the ratio of PC to SC was constant. The lower values of PC and SC were taken as 200 mg (threefold of 60 mg) and 40 mg, respectively, to get 0.2% *w/w* as final strength (2 mg/g). Similarly, higher levels of PC and SC were selected as 300 mg and 120 mg (threefold of 40 mg), respectively.

In addition, the best fit and generated mathematical models were chosen by equating various statistical parameters, such as a prototype F value, *p* value, and regular, adjusted, and predicted correlation coefficient (r^2^). Polynomial equations were generated, while optimization produced three-dimensional surface and contour graphs [28]. The suitability of the model was validated using *p* and F values, whereas the optimization process was validated using the individual (di) and overall desirability function (Di). Desirability is an objective numerical function approaching to 1. The desirability parameter equal to 1 justifies the best fit of the model and optimization method (factorial design) under set conditions of constraints and goal. Its values may be obtained between zero (poor) and 1 (ideal and best fit), and the maximum value indicates the closeness of the predicted and observed values. Zero value indicates unfit of the model applied. Statistically, “Di” is a simultaneous objective and geometrical mean functions of all studied responses (n), which depends upon constraints and goals (maximum, minimum, in range, and target) set for optimization (as shown in Equation (3)). Table 2 summarizes details of independent (X_1_ and X_2_) and dependent variables (Y_1_ to Y_4_).

Di = (d_1_. d_2_. …..d_n_) = (II_ii_ = _1_ di)^1/n^ (3)

This value depends upon several factors, such as (a) number of studied variables, (b) importance opted (+, ++, +++), (c) selected models (linear, quadratic, and others), and (d) goal (targeted, minimum, maximum, and in range). However, zero value of desirability function indicates failure of the selected model during optimization or of the variable as described before. A value near to 1 represents the best fit of the model in the optimization process, whereas a value near to 0 represents a poor fit of the model.

### 2.6. Evaluation of Software Suggested Nine Formulations

Several formulations comprised of PC and SC were prepared and characterized for vesicle size, % EE, % DR, and permeation flux. The optimized ELs and liposome formulations were evaluated and compared against the drug solution (DA solution).

#### 2.6.1. Morphological Assessment

The optimized formulation ODEL1 and placebo were visualized under SEM (Carl Zeiss, EVO43, SEM, Jena, Germany). The sample (2–3 drops) was placed on a glass coverslip and left for air drying (overnight). The samples were completely dried and then fixed on a double adhesive tape of copper grid. To make it electrically conductive, the samples were coated with gold using a coater. Finally, the coated samples were visualized for three-dimensional architecture of ELs at varied resolution.

#### 2.6.2. Drug Release Profile

The prepared formulations (DEL1 to DEL9, liposome, ODEL1, ODEL2, and DS) were investigated for in vitro drug release pattern using a dialysis membrane (molecular cutoff of 12–14 KDa, Himedia Labs). The test sample (2 mL) was placed in the membrane (served as donor), and tied from both ends using a clip. The receptor beaker was filled with PBS (200 mL, pH 7.4) and stirred at 100 rpm (using inert Teflon coated magnetic beads) and 37 ± 1 °C. Sampling (3 mL) was carried out at varied time points (0.5, 1, 2, 4, 8, 12, 16, 20, and 24 h), followed by replacement with fresh medium (equal volume). The concentration of the released drug was estimated by spectrophotometer at an absorption wavelength of 220 nm.

#### 2.6.3. Permeation Flux: Ex Vivo Study

Ex vivo studies were conducted using abdominal skin of albino rats (body weight of 250 ± 20 g and 6–8 weeks old male) issued from the College of Pharmacy, King Saud University, Riyadh, Saudi Arabia (approved ethical No: KSU-SE-20-64 dated on 02-12-2020). The study was carried out following the guideline for animal care and use of laboratory animals (NC3Rs, ARRIVE guidelines). Transdermal permeation of the formulations (DEL1 to DEL9, liposome, ODEL1, ODEL2, and DS) was assessed using a Franz diffusion cell. Rats were issued from the Institute ethical committee (College of Pharmacy, King Saud University, Riyadh, Saudi Arabia). Rat skin was obtained from the Institute animal house, and hairs were shaved using an electric shaver. The skin was made free from fatty debris and unwanted adhered tissues [29]. The skin was placed between both chambers (donor and receptor) in such a way that the dermal side faced PBS medium (pH 7.4) and the epidermis faced the loaded sample (equivalent to 20 µg of the drug). The release medium was stirred using beads (100 rpm) at 37 ± 1 °C. Furthermore, sampling was performed at varied time points (0.5, 1, 2, 4, 6, 10, 12, 16, 20, and 24 h) and the drug concentration was estimated using validated HPLC method at 220 nm. Permeation parameters (permeation flux, enhancement ratio, and cumulative amount of the drug permeated) were calculated. The study was carried out in triplicate to obtain mean and standard deviation (SD). Drug deposition was studied after completion of the permeation study using the same skin sample. The adhered sample was removed from the surface and then the skin tissue was sectioned into small pieces. The tissue was kept in a beaker containing methanol–chloroform (1:2) to extract the drug by stirring under a magnetic stirrer for 4 h. The tissue was filtered and the filtrate was analyzed for the drug content. The study was repeated for mean and SD values.

### 2.7. Hemocompatibility Study: Biosafety Assessment

It was essential to assess hemocompatibility of optimized ODEL1, ODEL placebo, PBS, and DS using rat’s blood. Normal saline and DW (distilled water) served as the negative and positive controls. The blood was collected in a blood collection tube containing anticoagulant from the retro-orbital portion of eyes, and centrifuged at 3000 rpm to separate erythrocytes [30]. Then, 4% RBCs (red blood cells) suspension was made in PBS (pH 7.4). Formulation (100 µL), 0.5 mL of 4% blood suspension, and 3.5 mL of PBS were gently mixed in a sterilized centrifuged tube. In the case of placebo, an equal volume (100 µL) of the sample was poured along with PBS and blood suspension. Similarly, saline and DW were the controls with same volume. The test tubes were sealed with paraffin film and incubated at 37 ± 1 °C for 2 h. After incubation, the test tubes were centrifuged to settle a pellet of lysed RBCs debris at the bottom, leaving a clear supernatant. The supernatant was removed (1 mL) and the released hemoglobin (Hb) was estimated at 540 nm using a spectrophotometer. The hemolysis caused by the positive control was considered as 100%.

### 2.8. Ex Vivo Vesicle–Skin Interaction Study Using Scanning Electron Microscopy (SEM)

To investigate the mechanistic aspect of vesicle permeation across the crystalline barrier of stratum corneum (SC), SEM was performed on treated rat skin and compared to the untreated (control) group. Group A served as a control (untreated), whereas group B, C, and D received treatment of DA solution, ODEL1, and ODEL2 and liposome, respectively. The treated area (1 cm^2^) was applied with formulation (0.2% *w/w*) and equal concentration of DA aqueous solution. The treated skin was allowed to interact for 2 h, and then the adhered sample (remained) was removed from the applied site using running water. The treated portion was excised from the site of application and left for air drying. The samples were subjected for SEM analysis and images were compared to the control group (untreated and DA solution). To visualize the perturbation and vesicle–skin interaction, images were captured at different magnifications. The surficial morphological changes were visualized under SEM.

### 2.9. Statistical Analysis

Experiments were replicated to obtain mean and standard deviation (*n* = 3). A value was considered statistically significant at *p* < 0.05. Graphical and statistical analyses were performed using a GraphPad (GraphPad prism, version 5.01, Inc., La Jolla, CA, USA), and Origin 6.1, v6, 1052 (B232) (Origin Lab Corporation, Northampton, MA, USA). Data of ANOVA (analysis of variance) were extracted from Design Expert^®^ during the optimization and validation process.

## 3. Results and Discussion

### 3.1. Prepared Elastic Liposomes and the Effect of Surfactants

Several ELs were prepared using varied ratio of PC to surfactants (PC: Tween 80, PC: Span 80, and PC: SC), as shown in Table 1. The purpose of selecting three different surfactants was based on hydrophilic, lipophilic, and ionic character of Tween 80, Span 80, and SC, respectively, which was expected to play a critical role on % EE, vesicle size, and elasticity of ELs. The result showed that there were remarkable differences on size, elasticity, PDI, and % EE when tailored with various types of surfactants and ratio of PC to surfactant, as shown in Table 1. Both hydrophilic Tween 80 and SC differ in terms of ionic nature, HLB value, and chemical structure (long hydrocarbon chain in Tween 80 and high HLB value ~15) [31]. However, Tween 80 caused relatively greater vesicle size range (178.1–335.6 nm), high PDI (0.28–0.47), low elasticity (15.2–36.3), and limited % EE (23.6–40.2%) as compared to SC, where these were in the range of 111.7–209.6 nm, 0.11–0.21, 35.2–66.7, and 47.8–77.4%, respectively. It is clear that low PDI values (0.11–0.21) represented a narrow vesicle size distribution and homogeneous nature of the tailored ELs using SC. Relative increment of surfactant to PC decreases the vesicle size in all formulations, which may be due to surfactant-based monomer assembly in vesicular structure. Lower content of lipid and high surfactant concentration may induce micelle formation as compared to high lipid and optimal surfactant [30,31]. The findings are in accordance to the reported value wherein acyclovir-sodium-loaded elastic liposomes were fabricated using Span 80 for transdermal delivery rationalizing the same fundamental cause [32]. Lipophilic Span 80 (HLB ~4.3) is easily internalized (improved solubilization) with the lipid component of the lipid bilayer in a vesicle, which, in turn, results in reduced size of the vesicles (156.6–212.7 nm) and % EE (~25%) as compared to highly hydrophilic Tween 80 (HLB ~15.0) [33,34]. Notably, % EE value first increases (from 10 to 30%), and then decreases at higher concentrations of Span 80 (40%), which may possibly be due to coexistence of the mixed micelles (generally at >15%) and vesicles at high content of surfactant [35,36]. On the other hand, hydrophilic Tween 80 and SC caused an increase in % EE with increase in the surfactant concentration. The hydrophilic nature of the drug and surfactant (Tween 80) attributed a larger vesicle size as compared to Span 80, which may be correlated with the possible effect of low free surface energy in Span 80. It is a well-known fact that the free surface energy decreases with the increase in hydrophobicity, which, in turn, results in decreased vesicle size and % EE [37]. Thus, the vesicle size of ELs depends upon various parameters, which may be taken into account during formulation design, such as (a) nature of surfactant (ionic, non-ionic, and amphiphilic), (b) types of hydrocarbon chain (saturated, unsaturated, branching, and length), (c) nature of head group (polar, charged or uncharged, and size), (d) concentration, (e) transition temperature, (f) temperature, (g) critical micellar concentration, and (h) solubility in lipid bilayer [35,36,37]. Results showed that SC-based formulations were found to have high % EE, optimum vesicle size, and high elasticity amongst them. Therefore, SC was selected as the suitable surfactant for further optimization using Design Expert (full factorial design at three levels of two factors).

### 3.2. Optimization Using Design Expert^®^

Design Expert^®^ (experimental tool) was used to optimize the most robust formulation by identifying factors, their levels, and relative importance. The tool also helped to identify possible interaction between factors against set responses. The technique optimizes factors at desired levels to get the most robust formulation as evidenced with several statistical parameters, models, and numerical objective functional parameters (desirability). In brief, the formulation DSC60 showed satisfactory outcomes (size, PDI, elasticity, and % EE) at 60% of PC and 40% of SC for transdermal delivery of 0.2 mg of desmopressin acetate. Therefore, higher and lower levels of X_1_ and X_2_ were set accordingly for developing formulation containing the drug as 2 mg/10 mL. Thus, Table 2 compiles detailed information of each factor at their three levels, constraints, goal, and models. The software generated a linear polynomial equation, expressed as Y = b_0_ + b_1×1_ + b_2×2_, where b_0_, b_1_, and b_2_ are the intercept and linear coefficients, respectively, for the response Y.

### 3.3. Desirability Function and Application

Desirability function is a numerical objective function applied for validation of the optimization process. Derringer and Suich developed this objective function to identify major factors affecting the optimization process under set constraints and importance given to the independent and dependent variables in order to comply these set of conditions for the responses [38]. The experimental tool suggested nine formulations (DEL1-DEL9) with given sets of X_1_ and X_1_, as shown in Table 2. Experimental values of vesicle size, PDI, % EE, % DR, and permeation flux were ranged as 123.9–171.7 nm, 0.14–0.53 (data not included in Table 2), 39.7–78.9%, 39.7–82.8%, and 0.111–5.71 µg/h·cm^2^, respectively. It is obvious from Table 2 that the values of vesicle size (Y_1_) were found to be progressively reduced (DEL1 to DEL3) with an increased concentration of X_2_ with respect to X_1_ (keeping constant). This may be due to edge-activator-mediated augmented emulsification and colloidal suspension formation. DEL7 demonstrated the highest value of vesicle size (271 nm) and the lowest value of permeation flux (1.37 µg/h·cm^2^), which may be attributed to the relatively high content of lipid than surfactant resulting in inefficient emulsification during the hydration step. Table 2 summarizes the predicted and observed values of Y_1_–Y_4_ for two optimized ODEL1 and ODEL2. The optimization process suggested two optimized formulations (ODEL1 and ODEL2) with desirability function values of 0.924 and 0.913, respectively. Detailed statistical parameters and generated polynomial linear equations for all of the responses (Y_1_ to Y_4_) are presented in Table 2. The observed values were found to be very close to predicted (in bracket) values, which suggested the best fit of the model.

### 3.4. Post-Optimization

#### 3.4.1. Vesicle Size (nm): Y_1_

**Figure 1:**
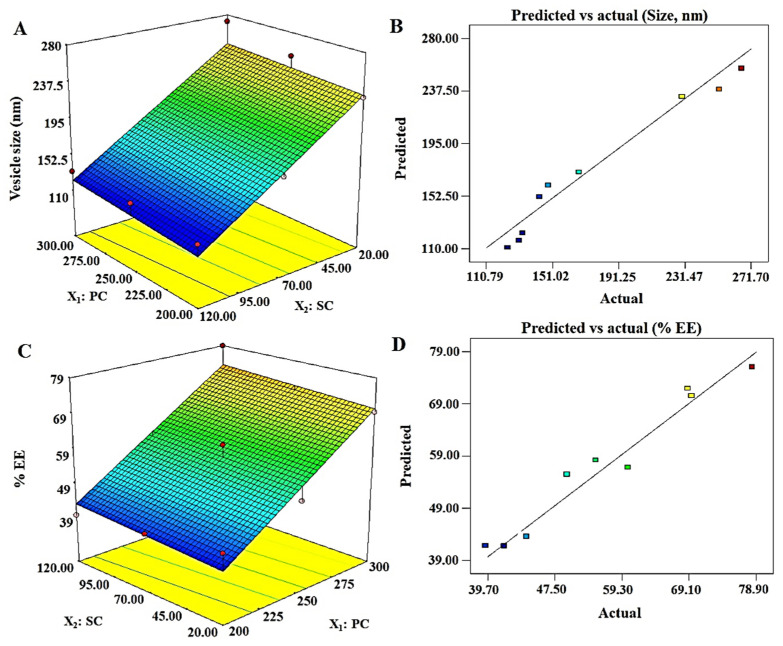
Software generated three-dimensional response surface plots and predicted vs. actual plots for the vesicle size (Y_1_) and % EE (Y_2_): (**A**) 3D response surface plot of Y_1_ illustrating an increment in size with increase in PC (phosphatidylcholine), whereas the size decrease with increase in SC (sodium cholate) content; (**B**) good correlation plot between predicted and actual values of vesicle size; (**C**) 3D response surface plot of Y_2_, which revealed a proportional relationship of both factors with the response; and (**D**) a good correlation of predicted versus actual values of % EE.

The vesicle size of ELs is a critical parameter intended for topical or transdermal delivery. This carrier has already been well established for delivery of hydrophilic and lipophilic molecules intended for local and systemic therapeutic effects. Moreover, ELs have their unique properties, which gained serious attention of formulations scientists and researchers for diverse uses. These features are the deformability, flexibility of the lipid bilayer membranes, stress-based adaptability, and sensitivity to the water gradient of skin [32,39]. This uniqueness (deformability and squeezing through smaller pores) is responsible for the vesicles ability to be permeated through the microscopic and sparsely dispersed numerous pores of the skin. The vesicle size values of DEL1 to DEL9 and generated polynomial linear equation for the response Y_1_ are presented in Table 2. The positive and negative sign of the term indicate synergistic and antagonistic effect of the factor on Y_1_. Vesicle size was ranged as 123.9–271.7 nm, and linear equation was Y_1_ = 177.71 + 5.85X_1_ − 61.07X_2_. Figure 1A, and B illustrated the response surface, and “predicted versus actual” plots of Y_1_, respectively. The linear equation was the best fit model as evidenced with ANOVA (analysis of variance) analysis, which can be justified with the low value of *p* (0.0032), high value of F (17.32), and r^2^ value of 0.989 (Table 3). The mathematical relationship of Y_1_ on factors (X_1_ and X_2_) revealed that the vesicle size significantly (*p* < 0.05) increases with increase in PC content, whereas it decreases with increase in SC. The value of adjusted regression coefficient (r^2^ = 0.9613) was very close to the predicted value (0.9601), suggesting a good agreement between them. Thus, an optimum formulation can be obtained by reducing PC content and increasing SC concentration in order to achieve the desired set goal (size at minimum ~123 nm).

Zeta potential values for Tween 80, Span 80, and SC-based formulations were in the range of −12 to −17.8, −15.3 to −21.8, and −28 to −34.9 mV, respectively. Higher values of zeta potential attained on the vesicle containing SC provided relatively stable vesicles due to repulsion existing between the vesicles (reduced aggregation) and suitability for improved dermal delivery. In all cases, net negative changes were found to be increased with an increase in surfactant concentration, whereas % EE was progressively decreased, which may be due to micellar formation at higher concentration of surfactant.

#### 3.4.2. % Entrapment Efficiency: Y_2_

The drug is hydrophilic and expected to be entrapped in the interior aqueous compartment of vesicles. Transdermal delivery of hydrophilic drug is challenging. Several hydrophilic drugs (insulin, 5-fluorouracil, and diclofenac) have been reported for improved dermal permeation due to maximized encapsulation in ELs [40,41,42]. Therefore, a constant amount of the drug (2.0 mg) was previously dissolved in hydration medium (10 mL). The result of Y_2_ for the formulations (DEL1–DEL9) is presented in Table 2. The % EE values ranged from 39.7 to 78.9 for DEL1–DEL9, as shown in Table 2. The % EE depends upon various factors, such as (a) physical state of surfactant, (b) types of surfactant, (c) HLB and (d) concentration of lipid, and surfactant. Here, the % EE was increased with increase in SC content, which may be correlated with the hydrophilic nature of SC, solid nature of SC, and desmopressin acetate. Literature suggested that the solid state nature of surfactant profoundly affects % EE, as observed with gel (Span 40 and Span 60), liquid (Span 80, Span 20, and Tween 80), and solid (sodium cholate and sodium deoxycholate) types of surfactants [43]. Gel surfactant being viscous reduces drug permeability across the lipid bilayer of vesicles as compared to liquid-based vesicular formulations. Therefore, Varshosaz et al. reported that Span 60- and Span 40-based niosomes showed high % EE of hydrophilic insulin as compared to Span-80-based formulation due to increased permeability in liquid-surfactant-based formulation [43]. Moreover, HLB value is another factor associated with % EE for a particular drug. High HLB-based surfactant (sodium cholate, HLB = 16.0) is suitable for maximum % EE of hydrophilic drug and vice versa. Thus, these combined effects may be a reasonable reason for increased % EE of desmopressin acetate in elastic liposomes containing SC. The finding was in good agreement with previous reports where 5-FU and diclofenac were maximally entrapped within elastic liposomes containing SC and, subsequently, 17-fold increased permeation flux was obtained across the rat skin [41,42]. A software generated mathematical equation is expressed as Y_2_ = 56.81 + 15.05X_1_ + 1.35X_2_, which established a relationship of the response (% EE) to the independent variables (X_1_ and X_2_) (Table 3). The polynomial equation for Y_2_ is well described, as evidenced with the statistical values of *p* (0.0004) and F (36.46). The 3D response surface plot (Figure 1C) was reviewed, which indicated that there is a significant increase in Y_2_ with increase in X_1_ and X_2_. Moreover, predicted and actual values were closely related as shown in Figure 1D. ANOVA analysis suggested that a closeness between the values of adjusted r^2^ (0.9891) and predicted r^2^ (0.9809) vindicated a good fit of the linear model for Y_2_. To obtain an optimized and most robust formulation, the concentration of X_1_ and X_2_ should be increased.

#### 3.4.3. % Drug Release: Y_3_

**Figure 2:**
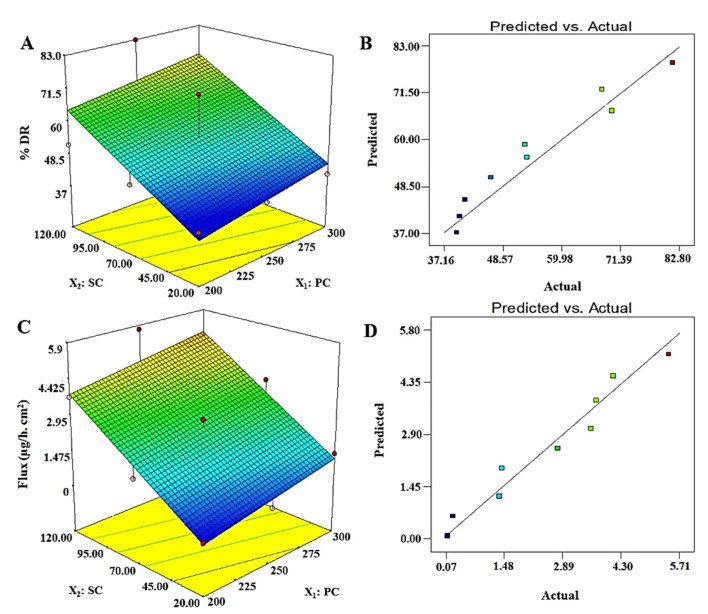
Software generated three-dimensional response surface plots and predicted vs. actual plots for % DR (Y_3_) and permeation flux (Y_4_): (**A**) 3D response surface plot of Y_3_ showed proportional increase in % DR with increase in PC and SC content, (**B**) a good correlation plot between predicted and actual values of % DR, (**C**) 3D response surface plot of Y_4_ which exhibited that Y_4_ is proportionally related with both factors (X_1_ and X_2_) and, (**D**) a good correlation of predicted versus actual values of Y_4_.

The results of % DR values are presented in Table 2 for DEL1 to DEL9, where the values ranged from 39.7 to 82.8%. Among them, DEL6 exhibited maximum release over a period of 12 h, which may be due to optimum content of X_1_ (PC = 250 mg) and X_2_ (120 mg). The generated polynomial equation was Y_3_ = 15.61 + 0.081X_1_ + 0.27X_2_, suggesting the linear model as the best fit model, which can be evidenced with the close relationship of adjusted and predicted r^2^ values (Table 3). The values of F (5.22) and *p* (0.048) further justified the best fit of the model for the optimization process. The 3D surface response plot of Y_3_ is illustrated in Figure 2A,B, wherein the % DR increases with increase in X_1_ and X_2_, which should be kept at an optimum level for the optimized formulation to achieve high desirability. Formulations DEL1, EL2, and DEL3 showed a slight increase in % EE due to probable chances of micelle formation at low lipid to surfactant ratio (1.66–10) at X_1_ = 200 mg. Similarly, formulations DEL4, EL5, and DEL6 showed significantly (*p* < 0.05) higher values of % EE due to vesicles formed at X_1_ = 250 mg (X_1_ to X_2_ ratio ranged as 2.01–12.5). Further increment in lipid, (DEL7, DEL8, and DEL9) revealed a reduction in % DR, which may be due to insufficient vesicles formed at X_1_ = 300 mg, precipitation of lipid (insufficiency of surfactant) (X_1_ to X_2_ ratio ranged as 2.5–15.0), and larger vesicles. In optimized formulation, ODEL1 composed of 285.0 mg of X_1_ and 115 mg of X_2_ was found to have % DR of 75.1%, which is closely related to the predicted value (72.28%), as shown in Table 3. Notably, the experiment was carried out in PBS (pH = 7.4) as desmopressin was reported chemically stable for a long time and considering physiological pH.

#### 3.4.4. Permeation Flux: Y_4_

Permeation of the drug across the skin remained a challenging task for several drug candidates due to the crystalline physiological nature of stratum corneum (SC) as a critical barrier. Therefore, desmopressin acetate was well explored and reported for transdermal delivery using physical methods, such as iontophoresis in a rat model [11,12,44,45]. However, no reports have been published using vesicular-based transdermal delivery of desmopressin acetate for safe and efficient delivery. In this study, a vesicle-based approach was implemented for transdermal delivery and results are presented in Table 2. Formulations DEL1–DEL9 showed that permeation flux values ranged from 0.111 to 5.71 µg/h·cm^2^ across a rat skin. A maximum permeation flux obtained was 5.71 µg/h·cm^2^ for DEL6, whereas the optimized ODEL1 and ODEL2 were found to have 5.3 µg/h·cm^2^ and 3.1 µg/h·cm^2^, respectively, as shown in Table 3. Thus, the enhancement ratio (ratio of the flux of formulation to the flux of pure drug solution) of ODEL1 and ODEL2 were found to be 7.57 and 4.43, respectively. In comparison to ODEL2, ODEL1 elicited relatively better permeation flux, which may be due to high entrapment efficiency (~79%) and smaller size (~118 nm). Cormier et al. reported non-depot delivery of 20 µg of desmopressin within 15 min using a microneedle array patch system across hairless guinea pig skin [14]. Our formulation (ODEL1) extended release of 19.92 µg of desmopressin acetate over a period of 240 min (16 times higher prolongation). Thus, transdermal elastic liposome formulation controlled and sustained the release profile of the drug for minimum plasma fluctuation (inter-subject variability) and prolonged therapeutic effect [44]. This controlled release may be attributed to the lipid bilayer as the first-rate controlling factor, and SC layer as the main-rate controlling physiological factor. There are several factors responsible in controlling permeation of the drug to the deeper dermal region or across SC layer of the skin. There were significant impacts of vesicle size and the surfactant concentration on the permeation flux, as shown in Table 2. From DEL1 to DEL3, the values of vesicle size were decreased due to increased surfactant concentration which results in increased permeation flux (from 0.111 to 3.71 µg/h·cm^2^). This may be correlated with the extended available surface area on size reduction and their augmented permeation across microscopic pores. A similar pattern was observed with other formulations (DEL4–DEL9). Figure 2D illustrated a linear relationship of permeation flux to the concentration of surfactant. The permeation flux was found to be higher on increasing the concentration of surfactant (X_2_). Therefore, an optimized formulation can meet the set goal by taking maximum concentration of surfactant and optimum content of lipid.

**Figure 3:**
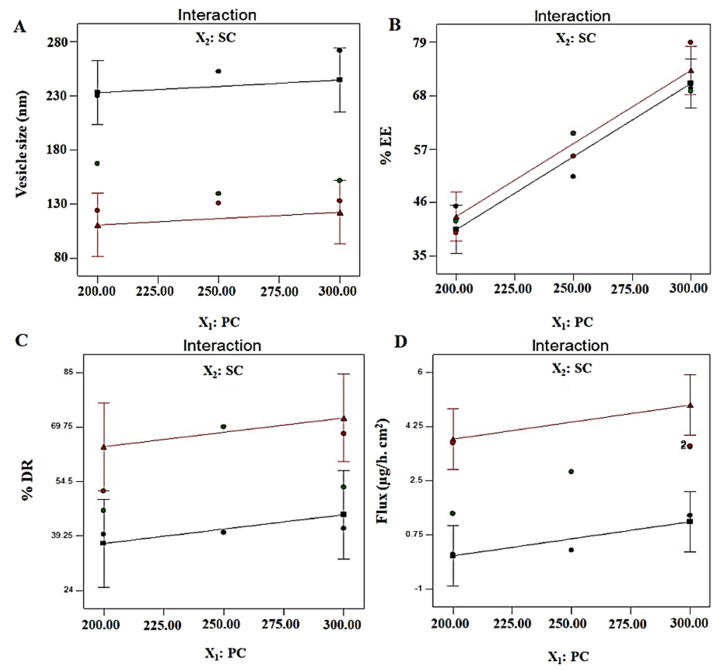
Interaction curve plots for (**A**) vesicle size (Y_1_), (**B**) % EE (Y_2_), (**C**) % DR (Y_3_), and (**D**) permeation flux (Y_4_). All responses exhibited parallel curves, suggesting no interaction between two factors (X_1_ and X_2_).

A generated polynomial equation was Y_4_ = 2.51 + 0.55X_1_ + 1.88X_2_, suggesting a linear relationship of the response with both studied factors (X_1_ and X_2_). ANOVA analysis report of statistical assessment confirmed the best fit of the model for optimization, as evidenced with the high value of F (15.99) and lower value of *p* (0.0039). Furthermore, a close relation of adjusted r^2^ and predicted values validated the best fit of the model. A surface response plot revealed a proportional relationship of Y_4_ with both factors to achieve an optimized formulation (Figure 2C). Moreover, predicted and observed values were in close agreement suggesting suitability of the model and optimization process (Figure 2D). Finally, it was required to analyze any possible interaction between the studied factors during optimization. There were no interactions observed (parallel lines to each other) between both factors against four explored responses (Y_1_, Y_2_, Y_3_, and Y_4_) (Figure 3). Conclusively, a detail of composition, predicted, and observed values of four responses for ODEL1 and ODEL2 is summarized in Table 3. These values (predicted and observed) are closely related, which indicated the best fit of the model.

**Figure 4:**
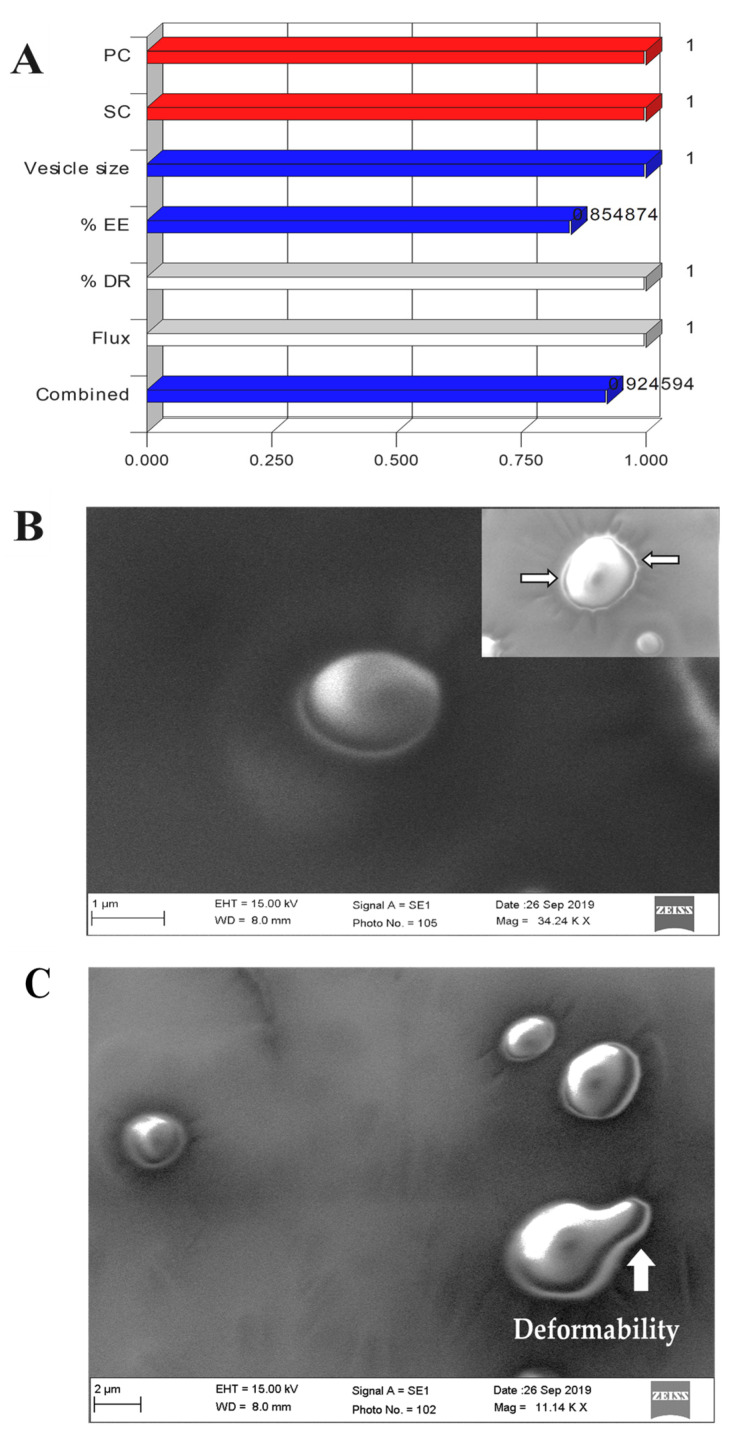
(**A**) Overall desirability bar graph and individual desirability of each factor and response, (**B**) representative images of scanning electron microscopy (SEM) of elastic liposome suspension (ODEL1), wherein inset represents elastic liposomes with round intact vesicles uniformly distributed in the colloidal suspension, (**C**) several spherical vesicles of ODEL2 exhibiting ultra-deformability (white arrow) passing through the microscopic pores of the membrane filter. Images were visualized for the sample partially passed through a membrane filter under vacuum.

Desirability function is an objective numerical function for the validation of the optimization process, taking independent factors and dependent factors under given sets of constraints. This numeric value varies between zero and one for individual, as well as overall desirability function. Overall desirability function depends upon various factors, such as number of factors and responses, given importance of each variable, model selected for optimization, and goal (target, in range, maximum, and minimum). Desirability function was used to identify prime factors and their levels affecting overall optimization in order to get maximum desirability for the most robust formulation. ODEL 1 and ODEL2 have overall desirability at 0.924 and 0.901, respectively, which are very close to one, suggesting the best fit of the model during optimization (Figure 4A).

### 3.5. Morphological Assessment

The developed elastic liposomes were scanned under SEM for morphological study and the result is portrayed in Figure 4B,C. The optimized ODEL1 showed that the vesicles were spherical in shape and free from aggregation (Figure 4C). Notably, the colloidal suspension of elastic liposomes was stable, as evidenced with homogeneously dispersed vesicles without signs of phase separation and aggregation scanned from various regions of the grid. Moreover, the inset of Figure 4B,C revealed the deformability of the elastic liposomes as the vesicle is squeezed out through the microscopic tiny pores (indicated by white arrow) of the membrane filter. This ultra-deformable capability of the elastic liposome membrane in response to physical stress (vacuum) resulted in a prompt change in shape, which is prudent to correlate for improved penetration into or through the skin for systemic drug access [27]. This can be correlated with mechanistic perspective of permeation across the rat skin. In addition, there was fracture or fragmentation of the vesicle while passing through the pore, which further vindicated its elastic nature and stability [32].

### 3.6. In Vitro Drug Release Kinetics and Comparison

**Figure 5:**
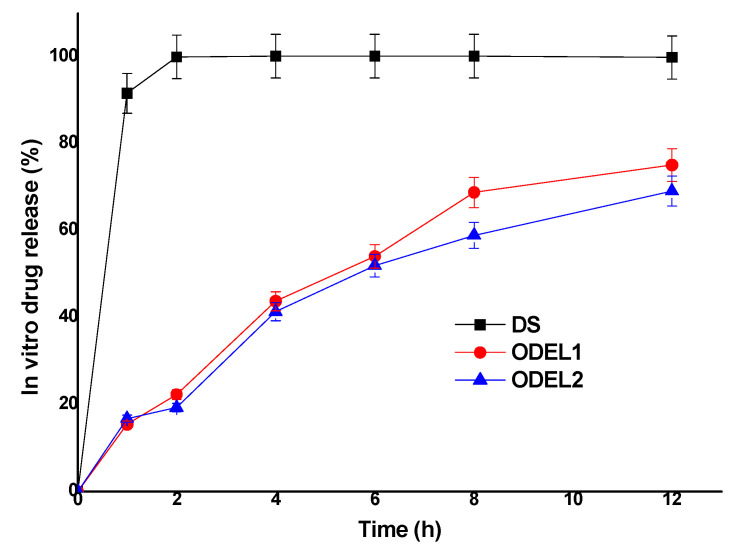
In vitro drug release pattern of two optimized ELs formulations (ODEL1 and ODEL2), as compared to drug solution (DS) over a period of 12 h.

In vitro drug release behavior of post-optimized formulations (ODEL1 and ODEL2) was studied as compared to free drug solution (DS) over a period of 12 h. The results are illustrated in Figure 5. As expected, free desmopressin acetate was soluble in release medium and found to be released about 99.8 percent within 2 h without any interaction with the dialysis membrane. Moreover, ODEL1 and ODEL2 exhibited slow and sustained release over the explored time points, as shown in Figure 5. ODEL1 and ODEL2 showed % DR of about 75.1% and 69.0%, respectively, at the end of 12 h. It is noteworthy that ODEL2 elicited relatively less release as compared to ODEL1, and a burst release of the drug at 1 h. This may be due to unentrapped drug and slightly less % EE as compared to ODEL1. Mathematical models applied to these two formulation confirmed Fickian diffusion as the release mechanism, and the zero order model was the best fit model, as evidenced with the obtained value of regression coefficient (r^2^ > 0.99) and diffusion coefficient (*n* = 0.21). Thus, the drug was slowly (sustained and controlled) released through the lipid bilayer membrane of ELs.

### 3.7. Skin Permeation Profiles and Drug Deposition

**Figure 6:**
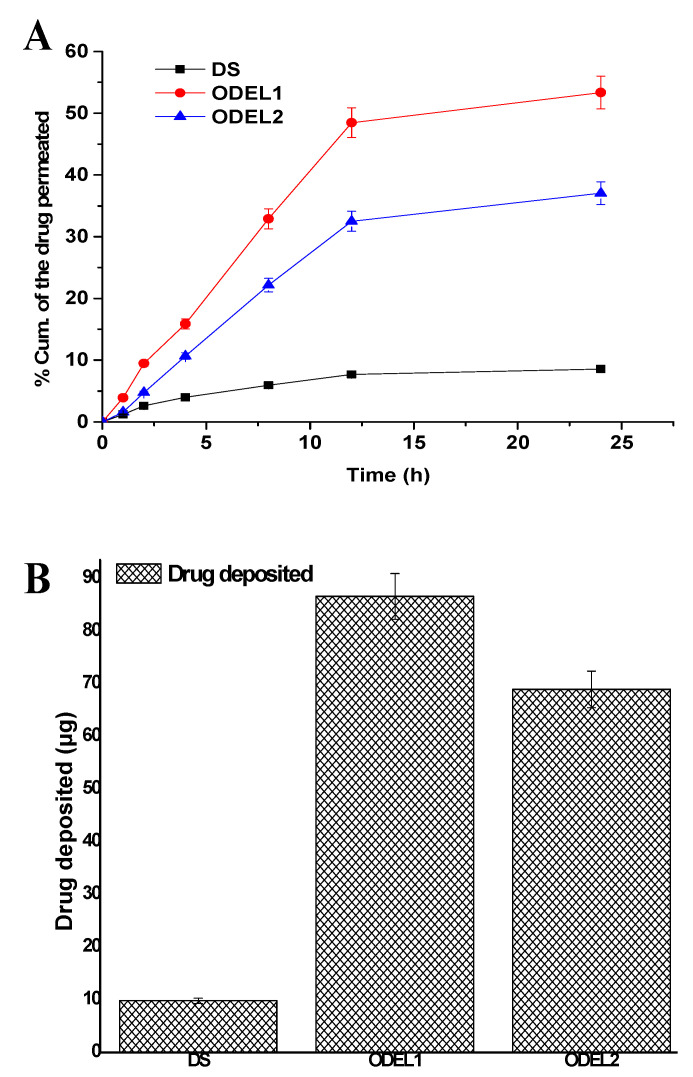
Ex vivo skin permeation and drug deposition studies of DS, ODEL1, and ODEL2: (**A**) % cumulative amount of desmopressin permeated at varied time points across rat skin, and (**B**) drug deposition into the skin after 24 h of permeation study. Data presented are mean ± SD (*n* = 3).

Formulations were examined for skin permeation flux and drug deposition, and results are portrayed in Figure 6A,B. The optimized formulation ODEL1 and ODEL2 exhibited augmented skin permeation across rat skin as compared to DS over a period of 24 h. ODEL1 and ODEL2 demonstrated % cumulative amount of drug permeation at 53.36% and 37.05%, respectively, at the end of 24 h, which are 6.23- and 4.32-fold higher than the DS. This can be due to the hydrophilic nature of the drug and its inability to permeate across the hydrophobic SC layer of skin. In contrast, drug-loaded ODEL1 and ODEL2 exhibited substantial permeation, which suggested that the encapsulated drug was successfully permeated through the epidermis via combined mechanisms (squeezing, deformability, reversible lipid extraction of SC, hydration effect, and surfactant-mediated permeation). Several sparsely distributed microscopic skin pores act as a permeability shunt as potential sites for improved permeation of the deformable bodies under the influence of transepidermal water gradients [32]. Moreover, the skin permeation flux values for DS, ODEL1, and ODEL2 were found to be 0.071, 0.53, and 0.31 µg/h·cm^2^, respectively. The calculated values of enhancement ratio for ODEL1 and ODEL2 (formulation flux over flux of DS) were 7.5 and 4.4, respectively, suggesting ODEL1-mediated permeation profiles were considerably high as compared to the drug solution.

Results of the drug deposition study are presented in Figure 6B. ODEL1, ODEL2, and DS revealed DD as 86.6 µg/cm^2^, 68.95 µg/cm^2^, and 9.86 µg/cm^2^, respectively. A maximum drug deposition is related to maximum permeation flux. This is why ODEL1 is associated with maximum permeation flux and maximum DD, as shown in Figure 6B. Theoretically, total body clearance and steady state concentration (C_max_) values of desmopressin acetate are reported as 0.0076 mL/h and 0.049 µg/mL, respectively [14]. A roughly estimated targeted flux value to deliver desmopressin acetate was ranged as 0.44 µg/h·cm^2^, considering 0.0076 L/h of total clearance (human), steady state plasma concentration of 4.0 µg/mL for a patient of 60 kg body weight, through an applied area of 0.5 cm^2^ on skin (FDA label, Minirin^®^, https://www.ferring.com/launch-of-minirin-2/ accessed on 2 June 2021) [46]. A clinical target dose of desmopressin acetate is 1–20 µg, which was achieved by transdermal delivery (guinea pigs) using a microneedle array technique (85% coated drug delivered 20 µg within 15 min) [14]. However, the technique (microneedle array) did not warrant in vivo efficacy due to challenges to monitor low-dose coated microneedle for its homogeneity, contamination (at the base of tip), and drug loss during penetration across the SC layer. Thus, the developed ODEL1 was found to have a permeation flux value within the clinical dose, and 19.92 µg was administered over a period of 4 h, suggesting controlled and sustained delivery and compliance with the clinical dose required. This would not only improve bioavailability, but high patient compliance and mitigated inter-subject variability on oral administration too.

### 3.8. In Vitro Hemolysis Study

**Figure 7:**
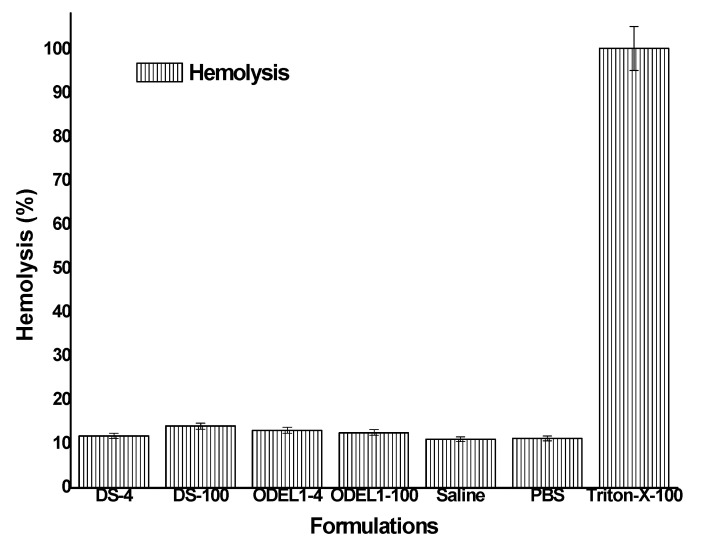
In vitro hemolysis study of various formulation incubated for 1 h.

In order to negate probable chances of hemolysis, it was requisite to assess in vitro hemolysis using rat blood. Hemolysis assay has been reported as a preliminary assessment for toxicity, reflecting physical damage to human cell membrane integrity [47,48]. The result is portrayed in Figure 7, where formulation could not exhibit significant hemolysis (*p* > 0.05) as compared to the negative control (saline). All of the formulations showed hemolysis in the range of 11.16–11.93%, whereas negative and positive controls elicited 11.13 and 100.38%, respectively. The hemolysis is indicative of the released hemoglobin as a result of hemolysis. Insignificant hemolysis caused by the formulation may be due to GRAS-based excipients used for the formulation development. No charge inducer or carrier was imposed on the vesicular system. Generally, positively charged polymer or positive charge imposed over nanoparticles are reported to have interaction with a negatively charged plasma membrane of erythrocytes, which subsequently results in hemolysis, as observed in chitosan-based nanoparticles causing ~223% hemolysis on human erythrocytes [49]. Elastic liposomes containing PC as major components are neutral, biocompatible and nontoxic to human cells. However, our formulations were biocompatible and suitable for transdermal delivery, as evidenced by the lower percent of hemolysis observed when exposed for 1 h.

### 3.9. Ex Vivo Vesicles–Skin Interaction Study Using SEM

**Figure 8:**
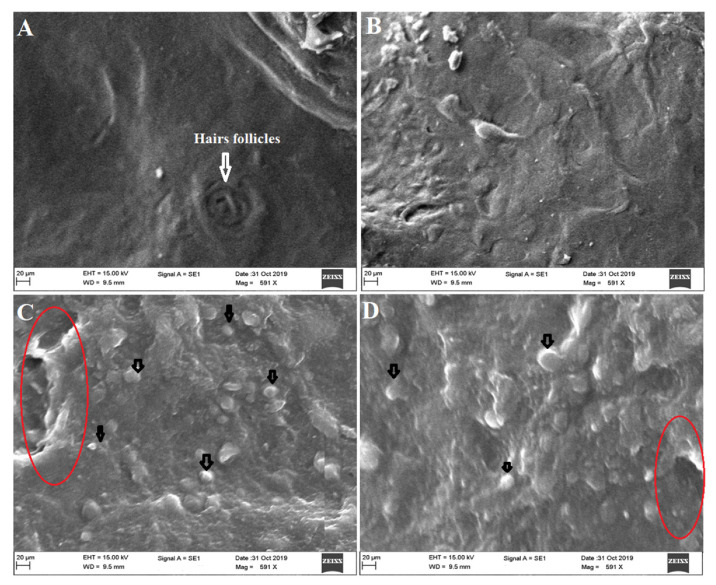
Ex vivo vesicles–skin interaction study using scanning electron microscopy exhibiting (**A**) untreated rat (control) skin exhibiting no structural changes in epidermal layer; (**B**) treating with DS showed hydrated skin without significant structural changes compared to formulation treated groups; (**C**) treating with ODEL1 revealed profound reversible structural changes in the epidermis (red encircled area) and interacting vesicle with the skin SC (black arrow); and (**D**) treating with ODEL2 formulation showed similar findings as ODEL1.

The ex vivo vesicles–skin interaction study ensured the mechanistic evaluation of permeation and interaction with the skin architecture. Images obtained from SEM study are presented in Figure 8A, wherein the control untreated group exhibited a normal morphology of the skin without any abnormalities (lesion, surgical mark, and inflammatory signs). The group treated with DS could not cause profound changes after topical application, as shown in Figure 8B. However, ODEL1- and ODEL2-treated groups exhibited substantial morphological changes in the epidermal layer (Figure 8C,D). Figure 8C,D illustrates visible interaction of the vesicle with SC layer of the skin for improved permeation. A red encircled area indicated that the vesicle attributed a significant perturbation of SC, resulting in augmented drug permeation as compared to DS. Moreover, there is no sign of drug precipitation or fractured vesicles post-application.

The epidermis layer consists hydrophobic SC layer, hair follicles, microscopic pores, and squamous tissue. Vesicles are well reported to permeate through the skin via several mechanisms, such as (a) squeezing out due to fluidity through pores and following hairs follicular pathways, (b) fusion with the skin lipid and (c) internalization with the skin tissue and, subsequently, SC lipid extraction (ethanol-mediated), (d) transepidermal hydration gradients, and (e) reversible changes of SC layer due to surfactant and lipid used in the formulation [32,50]. Furthermore, there are several factors that directly or indirectly influence the drug permeation across the skin, such as physicochemical properties of the drug, skin condition, types of dosage form, application area, dose and dosing frequency, and carriers (chemical and physical).

## 4. Conclusions

Considering in vitro findings and the results of ex vivo parameters, followed by vesicle–skin interaction, the drug-loaded elastic liposomes delivered a target dose of desmopressin acetate transdermally. The proposed elastic liposome improved drug loading, skin permeation, flux, and drug deposition as compared to drug solution. The carrier was capable of achieving targeted flux for therapeutic efficacy on transdermal delivery. The optimized formulation ODEL1 achieved targeted skin permeation flux of desmopressin acetate on transdermal application, exhibiting efficient and therapeutic drug delivery. Skin permeation flux and drug deposition values were significantly (*p* < 0.05) high as compared to the drug solution, which is prudent to correlate with the high deformability and elastic nature of elastic liposomes. Furthermore, the result of hemocompatibility assessment ensured the safety aspect of the developed formulation for transdermal application. The vesicle–skin interaction study corroborated a mechanistic perspective of the drug-loaded elastic liposomes permeated across the rat skin after topical application. The studied approach may be a new strategy to deliver desmopressin with minimum variability and maximum bioavailability. Conclusively, the study was a suitable alternative to oral, nasal, and physical methods of drug delivery to control diabetes insipidus, enuresis, hemophilia A, and von Willebrand’s diseases.

## Figures and Tables

**Table 1:** Composition and characterizations of desmopressin acetate loaded elastic liposomes.

Formulation Code	* PC: S Ratio (%)	Surfactant Type	Hydrating Media	Mean Size (nm)	** PDI	Elasticity	% EE
DELT90	90 (90:10)	Tween 80	PBS	335.6 ± 22.81	0.47	15.2 ± 2.1	23.6 ± 2.8
DELT70	70 (70:30)	Tween 80	PBS	203.9 ± 10.02	0.36	27.1 ± 2.7	32.6 ± 3.1
DELT60	60 (60:40)	Tween 80	PBS	178.1 ± 9.54	0.28	36.3 ± 4.2	40.2 ± 4.8
DELS90	90 (90:10)	Span 80	PBS	212.7 ± 23.22	0.47	25.8 ± 2.2	28.8 ± 5.1
DELS70	70 (70:30)	Span 80	PBS	169.5 ± 11.34	0.41	39.9 ± 3.7	53.8 ± 5.9
DELS60	60 (60:40)	Span 80	PBS	156.6 ± 10.54	0.23	58.0 ± 2.7	24.7 ± 6.2
DSC90	90 (90:10)	SC	PBS	209.6 ± 18.5	0.21	35.2 ± 5.7	47.8 ± 4.6
DSC70	70 (70:30)	SC	PBS	149.8 ± 14.8	0.21	58.2 ± 6.8	73.9 ± 7.1
DSC60	60 (60:40)	SC	PBS	111.7 ± 9.09	0.11	66.7 ± 8.1	77.4 ± 6.9
Liposome	PC: C: Span 80 (75: 10: 15)		PBS	276.8 ± 20.7	0.29	11.1 ± 0.5	32.0 ± 1.1

Values are represented as mean ± SD (*n* = 3), % EE = percent entrapment efficiency, ** PDI = polydispersity index, * PC: S = phosphatidylcholine, and S = surfactant, PBS (pH 5.5) = (0.1 M HCl and 0.154 M sodium chloride), C = cholesterol, SC = Sodium cholate.

**Table 2:** Combination of levels, independent, and dependent variables for elastic liposomes (ELs) loaded with desmopressin acetate.

Independent Variables	Levels					
	Low		Middle		High	
	Coded	Actual	Coded	Actual	Coded	Actual
X_1_: PC (mg)	−1	200	0	250	+1	300
X_2_: SC (mg)	−1	20	0	70	+1	120
Dependent Variables		Constraints				
		Low	High	Goal	Model	
Y_1_: Vesicle size (nm)		123.9	271.7	Minimum	Linear	
Y_2_: % EE		39.7	78.9	Maximum	Linear	
Y_3_: % DR		39.7	82.8	In range	Linear	
Y_4_: Permeation flux (µg/h·cm^2^)		0.111	5.71	In range	Linear	
Combination levels of independent variables and their responses						
Formulation Code	X_1_	X_2_	Y_1_	Y_2_	Y_3_	Y_4_
DEL1	200	20	229.8	45.2	39.7	0.111
DEL2	200	70	167.2	42.1	46.3	1.43
DEL3	200	120	123.9	39.7	51.8	3.71
DEL4	250	20	252.3	51.3	40.2	0.247
DEL5	250	70	139.5	60.2	69.7	2.78
DEL6	250	120	130.7	55.4	82.8	5.71
DEL7	300	20	271.7	69.5	41.3	1.37
DEL8	300	70	151.5	68.9	52.9	3.58
DEL9	300	120	132.8	78.9	67.8	3.61

**Table 3:** Summary of statistical analysis (3^2^ factorial design).

Model Coefficients	Responses			
	Y_1_	Y_2_	Y_3_	Y_4_
B_0_	177.71	54.81	54.72	2.50
B_1_	5.85	0.301	4.03	+0.5515
*p*-value	0.5949	0.0001	0.3914	0.1631
F-value	0.32	72.34	0.8528	2.52
B_2_	−61.07	0.027	13.53	1.88
*p*-value	0.0011	0.4744	0.02	0.0016
F-value	34.33	0.5821	9.6	29.49
Model Statistics				
r^2^	0.9898	0.9972	0.9963	0.9927
Adjusted r^2^	0.9613	0.9891	0.9813	0.989
Predicted r^2^	0.9601	0.9809	0.9706	0.981
Model F-value	17.32	36.46	5.22	15.99
Model *p*-value	0.0032	0.0004	0.048	0.0039
Observed/(predicted) Values				
Optimized	Y_1_	Y_2_	Y_3_	Y_4_
ODEL1 (X_1_ = 285 and X_2_ = 115 mg)	118.7 (122.49)	78.9 (73.21)	75.1 (72.28)	5.3 (0.58)
ODEL 2 (X_1_ = 272 and X_2_ = 100 mg)	131.9 (127.85)	74.13 (73.09)	69.0 (71.02)	3.1 (0.29)
Polynomial Equations for Each Response				
Y_1_ = 177.71 + 5.85X_1_ − 61.07X_2_				
Y_2_ = 56.81 + 15.05X_1_ + 1.35X_2_				
Y_3_ = 15.61 + 0.081X_1_ + 0.27X_2_				
Y_4_ = 2.51 + 0.55X_1_ + 1.88X_2_				

Y_1_ = vesicle size; Y_2_ = % entrapment efficiency; Y_3_ = % drug release; Y_4_ = flux; r^2^ = regression coefficient.

## Data Availability

Not applicable.
